# Genetic diversity and transmission patterns of *Echinococcus granulosussensu stricto* among domestic ungulates of Sardinia, Italy

**DOI:** 10.1007/s00436-021-07186-9

**Published:** 2021-06-19

**Authors:** Naunain Mehmood, Giorgia Dessì, Fahad Ahmed, Gaelle Joanny, Claudia Tamponi, Maria Grazia Cappai, Antonio Varcasia, Antonio Scala

**Affiliations:** 1 Department of Zoology, University of Sargodha, Sargodha, Pakistan; 2 Dipartimento Di Medicina Veterinaria, Università Degli Studi Di Sassari, Via Vienna, 2, 07100 Sassari, Italy

**Keywords:** Cystic echinococcosis, *Echinococcus granulosus s.s.*, Genetic diversity, Haplotypes, Sardinia

## Abstract

Cystic echinococcosis (CE), a parasitic zoonosis of public health and economic concern, is highly endemic in Sardinia, Italy. The study involved examining the intraspecific variability and demographic structure of *Echinococcus granulosus* *sensu stricto* (*s.s.*) in common hosts of this parasite. Molecular surveillance included the fragment amplification of a partial mitochondrial gene, *cox1* (750 bp), for a total of 69 isolates derived from sheep (*n* = 52), cattle (*n* = 11), pigs (*n* = 4), and goats (*n* = 2). It was ascertained that *E. granulosus s.s.* was the primary agent of infection among these ungulates and G1 genotype was highly prevalent (79.71%). Considerable intraspecific variation was found, revealing the existence of 22 haplotypes with relatively high haplotype (0.8555 ± 0.033) and low nucleotide diversities (0.00281 ± 0.00030). Population demographics indicated an expanding parasitic population signifying negative deviation from neutrality indices. Little genetic differentiation was found between the subpopulations of *E. granulosus s.s.* in the island. Moreover, the geographic dispersal of genotypes G1 and G3 also indicated similarity between Sardinian and mainland *Echinococcus granulosus s.s*. populations reaffirming the sympatric occurrence and efficient transmission of G1 and G3 genotypes. Molecular survey of CE has the potential to yield baseline information on the infective genotypes among the intermediate hosts and helps in devising suitable control strategies for curtailing the disease.

**Supplementary Information:**

The online version contains supplementary material available at 10.1007/s00436-021-07186-9.

## Introduction

Cystic echinococcosis (CE) is a globally widespread zoonosis caused by the larval stages of a tapeworm *Echinococcus granulosus* *sensu lato* (*s.l*.). It is listed among WHO-neglected diseases for which control strategies are suggested (Romig et al. 2015). Dogs and wild canids are usually the definitive hosts which harbor the adult stages of this parasite. Eggs are shed in feces of the definitive host and dispersed in the environment, where they can be picked up by a wide range of intermediate hosts and humans where the eggs can develop to larval stage (metacestode) forming hydatid cysts in internal organs and cause CE (Deplazes et al. 2017; Thompson 2017). Globally, CE is of major health significance due to indirect revenue losses incurred from human morbidity and mortality and direct economic losses to livestock industry because of offal condemnation (Eckert and Deplazes 2004; Budke et al. 2006; Battelli 2009).

Taxonomy of the genus *Echinococcus* has remained a challenging issue for decades due to striking intraspecific genetic diversity, morphology, life cycle, and host range differences (Romig et al. 2015). Thus, the taxonomy of this cryptic species complex has experienced perpetual revisions on the basis of adult morphological traits and genetic studies involving mitochondrial and nuclear genomes (Saarma et al. 2009; Nakao et al. 2013). Initial strain description based on intraspecific variability at mitochondrial level was provided by Bowles et al. (1992). Subsequent studies, relying on the partial and complete mitogenome analysis and nuclear genomic studies, aimed to clarify the species composition within *E. granulosus* *sensu lat*o. Current species now include the most common *E. granulosus* *sensu stricto* (G1 and G3 genotypes), *Echinococcus equinus* (G4 genotype), *Echinococcus ortleppi* (G5 genotype), *Echinococcus canadensis* (G6–G10 genotypes), and *Echinococcus felidis* (lion strain). The taxonomic status of genotypes G6/G7 and G8/G10 is still under dispute (Nakao et al. 2015; Romig et al. 2015; Laurimäe et al. 2018). Genotype G2, which was initially regarded as a distinct genotype (Bowles et al. 1992), was recently established to be a part of G3 (Kinkar et al. 2017). Therefore, G3 may be underrepresented due to erroneous allocation of CE cases to G2 (Kinkar et al. 2018a). Furthermore, G3 genotype, which was initially suggested to be buffalo specific (Bowles et al. 1992), was subsequently identified in multiple intermediate hosts like sheep, cattle, goats, camels, and wild boars implying the transmission potential of G3 beyond buffalo (Sharbatkhori et al. 2011; Laurimäe et al. 2019; Mehmood et al. 2020). Relatively high prevalence of the G3 strain is being recorded in Italy and Sardinia compared to other European and Mediterranean countries indicating its spread beyond the Indian region (Capuano et al. 2006; Busi et al. 2007; Kinkar et al. 2018a).

In Italy, CE is widespread and present in Sardinia (Varcasia et al. 2020). Sardinia is the second largest Mediterranean island and hosts more than 40% of the entire national sheep stock (Conchedda et al. 2010). Studies on the intermediate hosts have revealed very high rate of infection in sheep ranging between 65.3% (Varcasia et al. 2020) and 75% (Scala et al. 2006) followed by cattle (41.5%), pigs (9.4%; Varcasia et al. 2006), and wild boars (3.7%; Varcasia et al. 2008). Among the prevalent species of the parasite, *E. granulosus s.s.* is the most widespread species of the complex found in all intermediate hosts (Varcasia et al. 2006). Previously, *E. equinus* and *E. canadensis* (G7 genotype) have been reported from the horses and pigs in Sardinia (Varcasia et al. 2006, 2008). Therefore, it is highly needed to understand the transmission patterns and regional segregation of *E. granulosus s.s.* in all intermediate hosts with special attention to endemic diffusion of CE in Sardinia. Thus, the current study aimed at molecular screening of *E. granulosus s.s.* from the different intermediate hosts (sheep, cattle, pigs, and goats) for appropriately estimating the prevalence of infective genotypes and their correct allocation on the basis of partial mitochondrial *cox1* marker. Moreover, the population structure analysis of *E. granulosus s.s.* genotypes circulating among animal hosts in Sardinia was also undertaken to highlight genetic and demographic patterns.

## Material and methods

A total of 70 hydatid cyst specimens were collected from the sheep (*n* = 52), cattle (*n* = 11), pigs (*n* = 4), and goats (*n* = 3) during routine meat inspection in different municipalities of Sardinia from 2012 to 2018. Cyst presence in the visceral organs was analyzed by visual inspection and palpation. Infested organs (liver/lungs) were transported to the laboratory of Parasitology and Parasitic Diseases, Veterinary Teaching Hospital, University of Sassari, for further processing.

DNA was extracted from either germinal layer or protoscoleces using a commercial DNA extraction kit (Roche Diagnostics, USA) following the instruction manual. DNA concentration was assessed by a NanoDrop™ Lite spectrophotometer (Thermo Fisher Scientific, MA). PCR amplification for the partial mitochondrial gene, *cox1*, was done using primer pairs described by Nakao et al. (2000). Purified PCR products were sent for bidirectional sequencing in both forward and reverse directions (ABI Prism 3100 Genetic Analyzer, Applied Biosystems).

All sequenced chromatograms were checked individually for base-calling errors on FinchTV viewer (Geospiza Inc., Seattle, WA, USA). Obtained sequences (750 bp) were subjected to multiple sequence alignment along the reference sequence (Nakao et al. 2000) using Clustal X2 program (Larkin et al. 2007). Aligned data for the sequences were exported to DnaSP 6 program (Rozas et al. 2017) in FASTA format to compute polymorphism (number of mutations, haplotype number, and frequency) among the population under study. Haplotypic network was inferred using PopArt software (Leigh and Bryant 2015). Population genetics analysis was done using Arlequin package 3.5 (Excoffier and Lischer, 2010) to estimate nucleotide and haplotype diversities, neutrality indices (Tajima’s D and Fu’s Fs) among the Sardinian intermediate hosts. A pairwise fixation index (Fst) among the *E. granulosus s.s.* populations from Sardinia and the other Mediterannean countries (Italy, Turkey, France, Spain, Tunisia, and Algeria) was computed to understand the degree of gene flow among these countries (Boufana et al. 2014, 2015a, 2015b; Kinkar et al. 2016, 2017, 2018b; Laatamna et al. 2019; Bonelli et al. 2020; Table S1). Pairwise divergence in each intermediate host population from Sardinia and maximum likelihood phylogenetic analysis were done by MEGA X software (Kumar et al. 2018). Reference sequences used to compute inter and intraspecific phylogenies were taken from Nakao et al. (2000) for *E. granulosus s.s.* (G1), Nakao et al. (2002) for *Echinococcus multilocularis*, Nakao et al. (2003) for *Taenia solium*, Nakao et al. (2007) for *E. multilocularis*, *Echinococcus vogeli*, *Echinococcus oligarthra*, *E. canadensis* (G6 and G7), and Bonelli et al. (2020) for *E. granulosus s.s.* (G3).

## Results

**Fig. 1:**
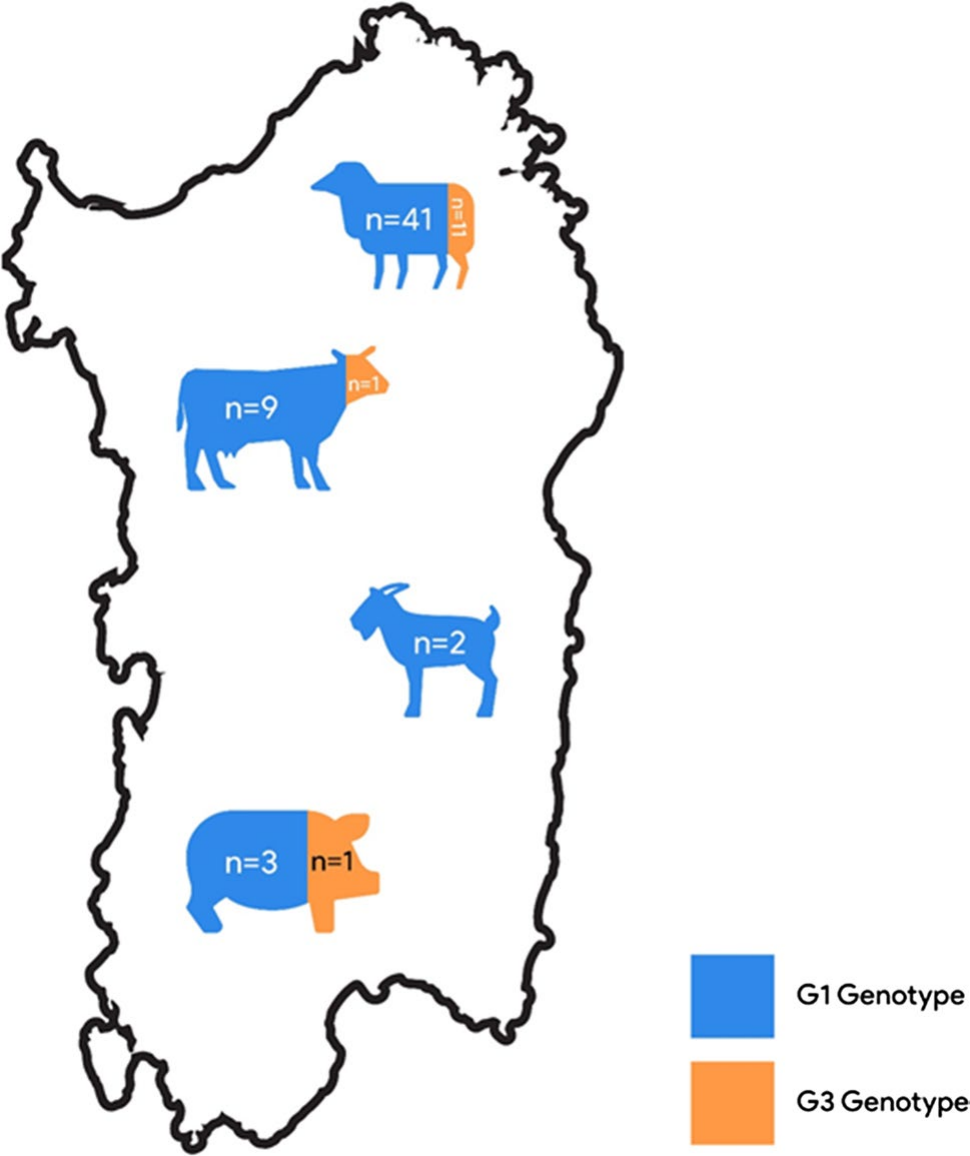
Map of Sardinia stating prevalence of G1 and G3 genotypes among the examined isolates (*n* = 69) form different intermediate hosts. Colors are indicating frequency of each genotype in the intermediate hosts

Successful amplification was achieved for 69 isolates out of 70 collected specimens; one goat isolate did not yield readable sequence and was, therefore, excluded from the molecular analysis. Obtained sequences were aligned with the reference sequence (Nakao et al. 2000) for genotypic and haplotypic assessments. G1 genotype was the predominant genotype appearing in 55 isolates (79.71%) belonging to sheep (*n* = 41, 78.84%), cattle (*n* = 9, 81.81%), pigs (*n* = 3, 75.00%), and goats (*n* = 2, 100%). G3 genotype was detected in 13 isolates originating from sheep (*n* = 11, 21.15%), pigs (*n* = 1, 25.00%), and cattle (*n* = 1, 9.10%) (Fig. 1).

**Fig. 2:**
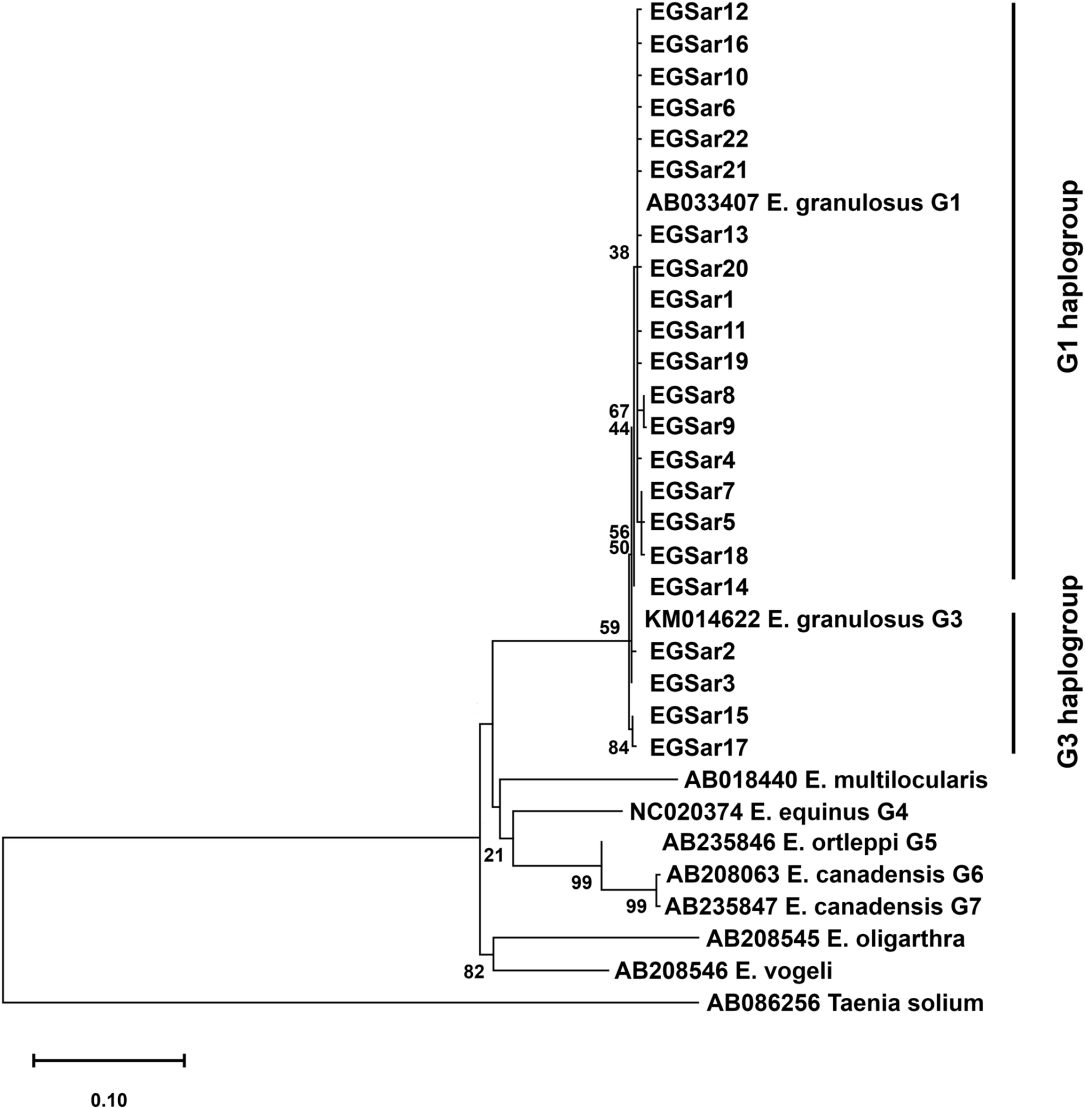
Phylogenetic tree based on maximum likelihood method indicating positioning of examined sequences with G1 and G3 genotype (*E. granulosus s.s.*) reference sequences

A total of 22 mutations were identified in 69 sequences at 22 segregating loci, of which 11 (50%) were parsimony informative. Among the total 22 nucleotide substitutions, 9 were non-synonymous (40.90%) and 13 were synonymous (59.10%). No indels or gaps were detected (Table 1). The nucleotide substitutions at the polymorphic loci were manifested by more transitions (*n* = 18) than the transversions (*n* = 3). A maximum likelihood (ML) tree was constructed for phylogenetic resolution of the obtained sequences which clearly positioned the obtained sequences among *E. granulosus s.s.* (G1 and G3 strains) reference sequences (Fig. 2). Degree of genetic divergence between the sequences was represented by horizontal branch lengths on the tree.

**Table 1:** Number, type of mutations, and pairwise divergence among *E granulosus s.s.* population of Sardinia

Host animals	No. of sequences analyzed	No. of mutations	Transitions/transversions	No. of polymorphic/segregating sites	Pairwise divergence (%)
Sheep	52	20	16/4	20	0.29
Cattle	11	8	7/1	8	0.24
Pig	4	4	3/1	4	0.27
Goat	2	0	0/0	0	0.00
Overall	69	22	18/3	22	0.28*

^*^Mean value

**Fig. 3:**
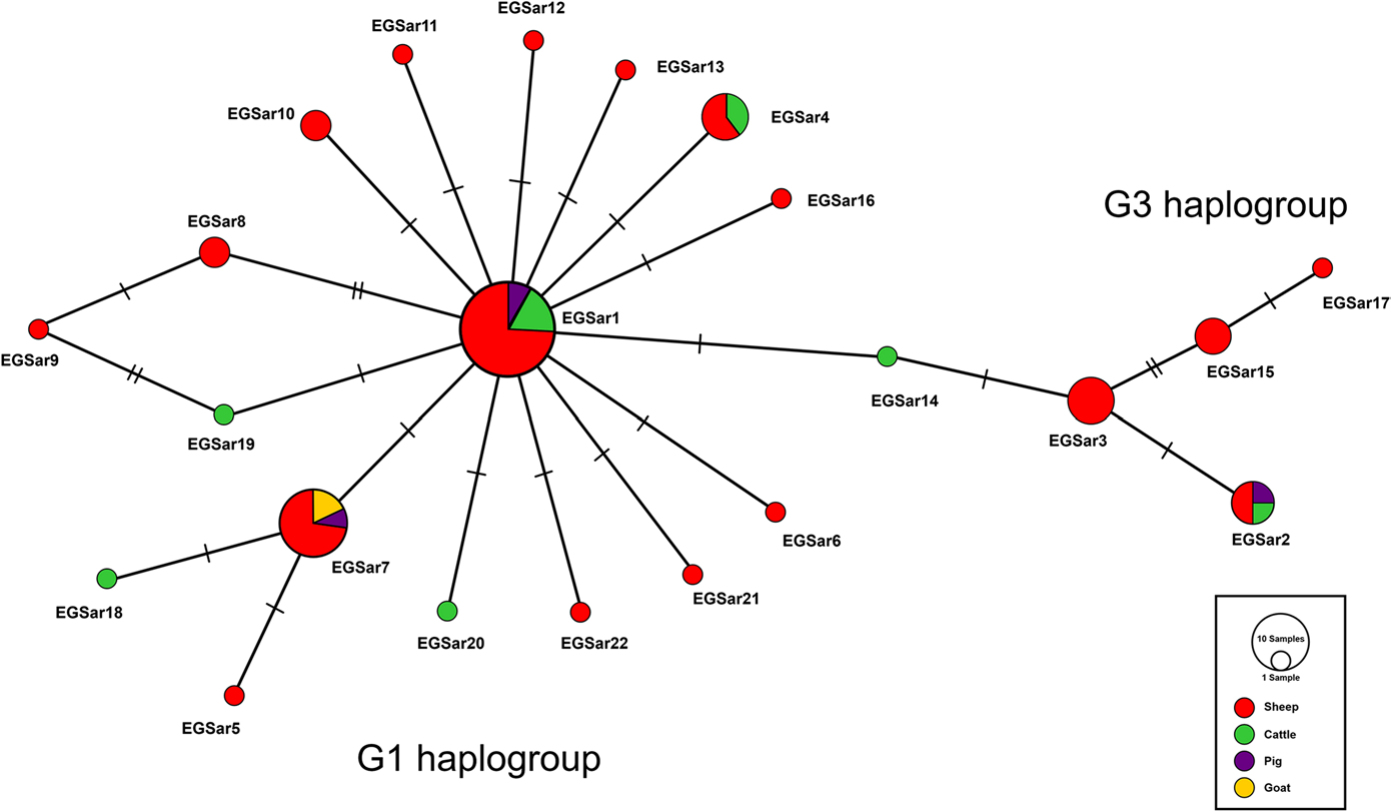
Haplotypic structure of *E. granulosus s.s.* genotypes G1 and G3 among domestic ungulates of Sardinia. Vertical lines correspond to the number of mutations between haplotypes and size of the circle indicates frequency of each haplotype (see also Table 2)

Haplotypic composition of the *E. granulosus s.s.* population demonstrated the occurrence of 22 haplotypes, among which 17 haplotypes (77.27%) grouped with G1 genotype whereas 4 microvariants (18.18%) were ascribed to G3 strain. A statistical parsimony network was constructed to discern genealogical relationship among the haplotypes which exhibited a star like configuration (Fig. 3). Clustered around a dominant haplotype, EgSar1, the network topology confirmed the presence of a common haplotype (33.33%) among Sardinian *E. granulosus s.s.* population (Table 2). The nucleotide sequence of EGSar1 was 100% identical to the dominant haplotype reported in earlier studies from Sardinia (Bonelli et al. 2020), Italy (Sgroi et al. 2019), UK (Boufana et al. 2015c), Tunisia (Boufana et al. 2014), and China (Ma et al. 2012). Fourteen unique G1 haplotypes were identified, of which 12 were singleton variants mainly characterized from sheep (*n* = 9). None of the goat isolates harbored the dominant G1 haplotype. The second most common haplotype, EgSar7 (15.94%), shared 100% similarity with microvariant reported earlier in Tunisia (Boufana et al. 2014). G3 haplogroup was only represented by 4 haplotypes which formed a small cluster separated by 2 or 3 mutational steps from the common G3 haplotype, EgSar3 (7.24%). G3 haplotypes displayed low nucleotide polymorphism and were identified from sheep, cattle, and pig isolates only. One haplotype, EgSar14, identified from cattle was shared among both G1 and G3 haplogroups with one mutational difference from both genotypes.

**Table 2:** Number of haplotypes and their prevalence in examined host populations

Genotype	Haplotype name	Number in the population	Prevalence (%)	Host animals	Accession number
G1	EGSar1	23	33.33	Sheep, cattle, goat, pig	MW243944
G3	EGSar2	4	5.79	Sheep, cattle, pig	MW243945
G3	EGSar3	5	7.24	Sheep	MW243946
G1	EGSar4	5	7.24	Sheep, cattle	MW243947
G1	EGSar5	1	1.45	Sheep	MW243948
G1	EGSar6	1	1.45	Sheep	MW243949
G1	EGSar7	11	15.94	Sheep, goat, pig	MW243950
G1	EGSar8	2	2.89	Sheep	MW243951
G1	EGSar9	1	1.45	Sheep	MW243952
G1	EGSar10	2	2.89	Sheep	MW243953
G1	EGSar11	1	1.45	Sheep	MW243954
G1	EGSar12	1	1.45	Sheep	MW243955
G1	EGSar13	1	1.45	Sheep	MW243956
G1/G3	EGSar14	1	1.45	Cattle	MW243957
G3	EGSar15	3	4.34	Sheep	MW243958
G1	EGSar16	1	1.45	Sheep	MW243959
G3	EGSar17	1	1.45	Sheep	MW243960
G1	EGSar18	1	1.45	Cattle	MW243961
G1	EGSar19	1	1.45	Cattle	MW243962
G1	EGSar20	1	1.45	Cattle	MW243963
G1	EGSar21	1	1.45	Sheep	MW243964
G1	EGSar22	1	1.45	Sheep	MW243965

Substantial variation in the sequences accounted for further population genetics analysis on the *E. granulosus s.s.* isolates from the Sardinian intermediate hosts. Overall high haplotype diversity within all host species (0.8555 ± 0.033) was observed along with low nucleotide diversity (0.00281 ± 0.00030), a feature characteristic of expanding populations. High haplotype diversity was demonstrated for cattle (0.8727 ± 0.0891), sheep (0.8620 ± 0.0363), and pig (0.8333 ± 0.2224) isolates. Nucleotide diversity values were quite low ranging from 0.002424 ± 0.001717 (cattle) to 0.002958 ± 0.001856 (sheep). Both haplotype and nucleotide diversities were zero for the goat isolates because of occurrence of a single haplotype and limited sample size (*n* = 2). Tests of neutrality, computed to delineate the demographic characteristics of populations, indicated a negative bias. Neutrality indices were negative (*D* =  − 1.72040, Fs =  − 15.600) for the whole population. Tajima’s *D* value was only significantly negative for sheep population (− 1.64449, *p* < 0.05) whereas Fu’s Fs statistic was significantly negative for sheep (− 10.53163, *p* < 0.05) and cattle (− 3.32340, *p* < 0.05) populations (Table 3). Tajima’s *D* is based on polymorphism among the alleles and the significant values indicate an excess of rare nucleotide site variants in comparison to what is expected under neutral model of evolution. The Fu’s Fs statistic, on the other hand, is based on the variant alleles within the population and the significant values signify population expansion and occurrence of rare haplotypes compared to what would be expected under neutrality. To understand gene flow patterns for *E. granulosus s.s.*, a pairwise Fst was calculated for different regional populations of the Mediterranean region and Sardinia. Low Fst values were exhibited for *E. granulosus s.s.* populations of Sardinia and Italy (− 0.01193) whereas highest Fst value was observed for Sardinia and Tunisia (0.07382, *p* < 0.05) (Table 4).

**Table 3:** Diversity and neutrality indices for *Echinococcus granulosus s.s.* form the intermediate hosts of Sardinia

				Diversity indices		Neutrality indices	
		No. of analyzed sequences	Hn	Hd ± SD	Nd ± SD	Tajima’s *D*	Fu’s Fs
Intermediate hosts	Sheep	52	18	0.8620 ± 0.0363	0.002958 ± 0.001856	− 1.64449*	− 10.53163*
	Cattle	11	7	0.8727 ± 0.0891	0.002424 ± 0.001717	− 1.49340	− 3.32340*
	Pig	4	3	0.8333 ± 0.2224	0.002778 ± 0.002330	− 0.78012	0.13353
	Goat	2	1	0.000 ± 0.0000	0.0000 ± 0.00000	0.00000	0.00000
	Overall	69	22	0.8555 ± 0.033	0.00281 ± 0.00030	− 1.72040	− 15.600

^*^Significant at *p* < 0.05

**Table 4:** Pairwise fixation index (Fst) for studied populations of *E. granulosus s.s.* for Sardinia and other Mediterranean countries

	Italy	Turkey	Spain	Tunisia	Algeria
Sardinia	− 0.01193	0.06537*	0.03276	0.07382*	0.06735*

^*^Significant at *p* < 0.05

## Discussion

Genetic diversity and population structure analysis of *E. granulosus s.s.* were evaluated on the basis of partial mitochondrial *cox1* gene. The *cox1* genotyping confirmed the presence of *E. granulosus s.s.* in all livestock species. Considering the widespread presence, it could be emphasized that *E. granulosus s.s.* was the primary species in disease etiology among the domestic ungulates of Sardinia. Molecular characterization revealed the existence of shared and unique haplotypes among the host animals indicating circulation and cross-transmission of *E. granulosus s.s.* between these intermediate hosts and the role of these ungulates in perpetuation of domestic cycle. *E. granulosus s.s.* is maintained in synanthropic cycles involving domestic herbivores which potentially harbor fertile cysts and maintain infection reservoir for dogs and, therefore, humans (Boufana et al. 2014).

G1 has cosmopolitan distribution (Kinkar et al. 2018c) and is regarded as the most prevalent strain across Mediterranean region (Bonelli et al. 2018), Europe (Casulli et al. 2012; Kinkar et al. 2016), China (Yanagida et al. 2012), South America (Laurimäe et al. 2016; Rojas et al. 2017), and Africa (Boufana et al. 2014) among all intermediate hosts including humans. Even though genotypes G1 and G3 are grouped together as *E. granulosus s.s*., distinctiveness among these strains is present at mitochondrial level (Kinkar et al. 2017; 2018a), but not when the nuclear genes are analyzed (Kinkar et al. 2017). G3 strain, also named as buffalo strain (Bowles et al. 1992), is less prevalent globally but commonly occurs in areas with large buffalo populations like Italy (Capuano et al. 2006; Busi et al. 2007), India (Sharma et al. 2013) and Pakistan (Mehmood et al. 2020; Muqaddas et al. 2020). Sharing common evolutionary trajectory, members of *E. granulosus s.s.* (G1 and G3 strains) occupy similar ecological niches around the globe but marked differences in prevalence of these genotypes could be linked to paleo-zoogeographic events and parasite’s life history. Phylogeographic routes based on Bayesian model point towards probable transmission of G3 from Asia into Europe (Kinkar et al. 2018a).

Twenty-two (22) haplotypes of G1–G3 complex were obtained in current molecular analysis forming two haplogroups. G1 was found as the principal strain (79.71%) infecting all host types. A multiple star-like configuration was observed among the *E. granulosus s.s.* isolates originating from sheep, cattle, pigs, and goats. Sheep harbored maximum number of haplotypes (*n* = 18), most probably because 75.36% isolates were derived from sheep. Alternatively, it could also be argued that sheep were the key hosts in shaping epidemiologic patterns for *E. granulosus s.s.* at Sardinia. High haplotype diversity (0.8555 ± 0.033) in congruence with low nucleotide diversity (0.00281 ± 0.0030) was manifested for Sardinia, an observation quite similar to other studies displaying genetic diversity and population expansion (Casulli et al. 2012; Yanagida et al. 2012; Boufana et al. 2014; Bonelli et al. 2020).

All subpopulations of *E. granulosus s.s.* exhibited an overall negative deviation from neutrality (*D* and Fu’s Fs). Sheep exhibited significantly negative values for these indices whereas cattle had negative values for both components out of which Fu’s Fs value was significant. Occurrence of rare polymorphic alleles was suggested by negative statistics and significant values alluded to past bottleneck events as a result of purifying selection and recent demographic expansion. Positive and non-significant bias from neutrality indicates low genetic polymorphism among populations that have undergone bottleneck as evident from lower number of haplotypes in pigs (*n* = 3) which demonstrated positive value (0.13353) for Fu’s Fs. It is important to mention that this positive outcome could be due to a small sample size which may partially account for detecting low polymorphism in pig specimens. Genetic differentiation estimates between different *E. granulosus s.s.* populations originating from the Mediterranean countries yielded very low Fst value among Sardinia and Italy (− 0.01193, *p* > 0.05) indicating higher gene flow. While comparing Sardinia with other Mediterranean countries, it was observed that none of the Fst value was negative; however, low Fst value for Spain and Sardinia implied sharing of alleles. Free gene flow because of geographical connectivity and absence of forces that lead to structuring of the populations is evident from the low Fst values.

To the best of our knowledge, the present manuscript describes for the first time G3 in pigs, with haplotype EGSar2. *Echinococcus granulosus s.s.* does not primarily target pigs (Paoletti et al. 2019); however, G1 genotype is reported from pigs in Sardinia (Varcasia et al. 2006; Bonelli et al. 2020) and other endemic areas (Casulli et al. 2012; Tigre et al. 2016; Laurimäe et al. 2019; Umhang et al. 2020). Fertile G1 hydatid cysts (69.2%) have earlier been detected in swine isolates. Pigs and sheep, in comparison with other intermediate hosts, have more defined role in CE epidemiology in Sardinia due to higher cyst fertility rates (Varcasia et al. 2006). Presence of a G3 variant in pig is probably a sign of host overlapping and expansion of host spectrum by G3 genotype; pigs are usually known to harbor G7 genotype (Romig et al. 2015). However, specific future investigations pertaining to swine population of Sardinia are needed to corroborate the role of pigs and their adaptability to different genotypes (G1, G7 and G3) of *E. granulosus s.l.* (Varcasia et al. 2006).

One haplotype, EGSar14, fell between G1 and G3 genotypes; according to original description of these strains by Bowles et al. (1992) in smaller *cox1* fragment of 366 bp, one diagnostic position was carrying nucleotide substitution similar to G3 genotype while the other position carried the nucleotide substitution as that of G1 strain. Both of these discriminating sites are present in gene fragment under study at positions 504 and 695 according to the reference sequence given by Nakao et al. (2000). Similar intermediate haplotypes have been identified in other studies at Turkey (Šnábel et al. 2009), Tunisia (Boufana et al. 2014), and China (Ma et al. 2015). All such haplotypes are mainly associated with G1 genotype because of diagnostic relevance of second position which entails more significance in strain identification (Šnábel et al. 2009). Recently, *nad5* mitochondrial gene is proposed to be a very good gene marker in discriminating G1 and G3 strains (Kinkar et al. 2018b); such haplotypes could be sequenced using *nad5* gene marker for correct identification of these isolates.

## Conclusion

The present study provides a compelling evidence on predominant involvement of *E. granulosus s.s.* in cystic echinococcosis on Sardinia island. Despite being an insular region, Sardinia is considered highly endemic for CE with sheep-dog cycle as the prominent synanthropic route for the transmission of this parasite. The current study highlighted substantial genetic variation at mitochondrial level (partial *cox1*) within the sheep and buffalo strains by reaffirming their expansion within the host populations. A G3 haplotype was also identified from pig indicating its incorporation into pig-dog cycle; however, data on cyst fertility is to be correlated with such observations to reach some concrete conclusion. This study also emphasizes the need for refining transmission dynamics for goats and pigs; further studies involving more isolates from goats and pigs must be carried out for identifying their role in disease epidemiology. This study has provided an insight into infective and prominent genotypes cycling within the intermediate hosts and would enable the authorities to devise suitable control strategies for this disease in this hyperendemic region for CE.

## Supplementary Information

Below is the link to the electronic supplementary material.

Supplementary file1 (DOCX 28 KB)

## Data Availability

Not applicable.
