# Supplementary material for: Genetic diversity and transmission patterns of Echinococcus granulosussensu stricto among domestic ungulates of Sardinia, Italy
Source: Parasitol Res. 2021 Jun 19;120(7):2533–42. doi: 10.1007/s00436-021-07186-9 (PMC8263412; doi:10.1007/s00436-021-07186-9)
Supplement: Supplementary file 1 — Supplementary file1 (DOCX 28 KB) [file 436_2021_7186_MOESM1_ESM.docx]

**Table S1**

Accession numbers for the sequences used for computation of Fst between Sardinia and the other Mediterranean countries

| Accession number | Country | Host | Reference |
| --- | --- | --- | --- |
| MG672283 | Algeria | human | Kinkar et al. 2018c |
| MG672284 | Algeria | human | Kinkar et al. 2018c |
| MG672285 | Algeria | human | Kinkar et al. 2018c |
| MG672286 | Algeria | human | Kinkar et al. 2018c |
| MG672287 | Algeria | human | Kinkar et al. 2018c |
| MG672288 | Algeria | human | Kinkar et al. 2018c |
| MG672289 | Algeria | human | Kinkar et al. 2018c |
| MG672290 | Algeria | human | Kinkar et al. 2018c |
| MG672291 | Algeria | human | Kinkar et al. 2018c |
| MG672292 | Algeria | human | Kinkar et al. 2018c |
| MG672293 | Algeria | human | Kinkar et al. 2018c |
| MG672128 | Algeria | human | Kinkar et al. 2018c |
| MG682544 | Algeria | human | Kinkar et al. 2018a |
| MG808282 | Algeria | cattle | Laataamna et al. 2019 |
| MG808283 | Algeria | cattle | Laatamna et al. 2019 |
| MG808284 | Algeria | cattle | Laatamna et al. 2019 |
| MG808285 | Algeria | cattle | Laatamna et al. 2019 |
| MG808286 | Algeria | sheep | Laatamna et al. 2019 |
| MG808287 | Algeria | sheep | Laatamna et al. 2019 |
| MG808288 | Algeria | sheep | Laatamna et al. 2019 |
| MG808289 | Algeria | sheep | Laatamna et al. 2019 |
| MG808290 | Algeria | sheep | Laatamna et al. 2019 |
| MG808291 | Algeria | sheep | Laatamna et al. 2019 |
| MG808292 | Algeria | cattle | Laatamna et al. 2019 |
| MG808293 | Algeria | sheep | Laatamna et al. 2019 |
| MG808294 | Algeria | sheep | Laatamna et al. 2019 |
| MG808295 | Algeria | sheep | Laatamna et al. 2019 |
| MG808296 | Algeria | sheep | Laatamna et al. 2019 |
| MG808297 | Algeria | sheep | Laatamna et al. 2019 |
| MG808298 | Algeria | sheep | Laatamna et al. 2019 |
| MG808299 | Algeria | sheep | Laatamna et al. 2019 |
| MG808300 | Algeria | cattle | Laatamna et al. 2019 |
| MG808301 | Algeria | sheep | Laatamna et al. 2019 |
| MG808302 | Algeria | sheep | Laatamna et al. 2019 |
| MG808303 | Algeria | sheep | Laatamna et al. 2019 |
| MG808304 | Algeria | sheep | Laatamna et al. 2019 |
| MG808305 | Algeria | sheep | Laatamna et al. 2019 |
| MG808306 | Algeria | cattle | Laatamna et al. 2019 |
| MG808307 | Algeria | cattle | Laatamna et al. 2019 |
| MG808308 | Algeria | sheep | Laatamna et al. 2019 |
| MG808309 | Algeria | sheep | Laatamna et al. 2019 |
| MG808310 | Algeria | sheep | Laatamna et al. 2019 |
| MG808311 | Algeria | sheep | Laatamna et al. 2019 |
| MG808312 | Algeria | sheep | Laatamna et al. 2019 |
| MG808313 | Algeria | sheep | Laatamna et al. 2019 |
| MG808314 | Algeria | sheep | Laatamna et al. 2019 |
| MG808315 | Algeria | sheep | Laatamna et al. 2019 |
| MG808316 | Algeria | sheep | Laatamna et al. 2019 |
| MG808317 | Algeria | sheep | Laatamna et al. 2019 |
| MG808318 | Algeria | cattle | Laatamna et al. 2019 |
| MG808319 | Algeria | sheep | Laatamna et al. 2019 |
| MG808320 | Algeria | cattle | Laatamna et al. 2019 |
| MG808321 | Algeria | sheep | Laatamna et al. 2019 |
| MG808322 | Algeria | cattle | Laatamna et al. 2019 |
| MG808323 | Algeria | sheep | Laatamna et al. 2019 |
| MG808324 | Algeria | cattle | Laatamna et al. 2019 |
| MG808325 | Algeria | sheep | Laatamna et al. 2019 |
| MG808326 | Algeria | sheep | Laatamna et al. 2019 |
| MG808327 | Algeria | sheep | Laatamna et al. 2019 |
| MG808328 | Algeria | sheep | Laatamna et al. 2019 |
| MG808329 | Algeria | sheep | Laatamna et al. 2019 |
| MG808330 | Algeria | cattle | Laatamna et al. 2019 |
| MG808331 | Algeria | cattle | Laatamna et al. 2019 |
| MG808332 | Algeria | sheep | Laatamna et al. 2019 |
| MG808333 | Algeria | sheep | Laatamna et al. 2019 |
| MG808334 | Algeria | sheep | Laatamna et al. 2019 |
| MG808335 | Algeria | sheep | Laatamna et al. 2019 |
| MG808336 | Algeria | sheep | Laatamna et al. 2019 |
| MG808337 | Algeria | sheep | Laatamna et al. 2019 |
| MG808338 | Algeria | sheep | Laatamna et al. 2019 |
| MG808339 | Algeria | sheep | Laatamna et al. 2019 |
| MG808340 | Algeria | sheep | Laatamna et al. 2019 |
| MG808341 | Algeria | cattle | Laatamna et al. 2019 |
| MG808342 | Algeria | cattle | Laatamna et al. 2019 |
| MG808343 | Algeria | cattle | Laatamna et al. 2019 |
| MG808344 | Algeria | cattle | Laatamna et al. 2019 |
| MG808345 | Algeria | cattle | Laatamna et al. 2019 |
| MG808346 | Algeria | sheep | Laatamna et al. 2019 |
| MG808347 | Algeria | sheep | Laatamna et al. 2019 |
| MG808348 | Algeria | cattle | Laatamna et al. 2019 |
| MG808349 | Algeria | sheep | Laatamna et al. 2019 |
| MK780826 | Sardinia | cattle | Bonelli et al. 2020 |
| MK780827 | Sardinia | cattle, pig, wild boar, human | Bonelli et al. 2020 |
| MK780828 | Sardinia | sheep | Bonelli et al. 2020 |
| MK780829 | Sardinia | sheep, pig | Bonelli et al. 2020 |
| MK780830 | Sardinia | sheep, human | Bonelli et al. 2020 |
| MK780831 | Sardinia | sheep | Bonelli et al. 2020 |
| MK780832 | Sardinia | sheep | Bonelli et al. 2020 |
| MK780833 | Sardinia | sheep | Bonelli et al. 2020 |
| MK780834 | Sardinia | sheep, goat, cattle, wild boar | Bonelli et al. 2020 |
| MK780835 | Sardinia | cattle | Bonelli et al. 2020 |
| MK780836 | Sardinia | sheep | Bonelli et al. 2020 |
| MK780837 | Sardinia | sheep | Bonelli et al. 2020 |
| MK780838 | Sardinia | sheep, goat | Bonelli et al. 2020 |
| MK780839 | Sardinia | sheep, cattle | Bonelli et al. 2020 |
| MK780840 | Sardinia | cattle | Bonelli et al. 2020 |
| MK780841 | Sardinia | domestic cat | Bonelli et al. 2020 |
| MK780842 | Sardinia | human | Bonelli et al. 2020 |
| MK780843 | Sardinia | human | Bonelli et al. 2020 |
| MK780844 | Sardinia | wild boar | Bonelli et al. 2020 |
| MK780845 | Sardinia | sheep | Bonelli et al. 2020 |
| MK780846 | Sardinia | sheep | Bonelli et al. 2020 |
| MK780847 | Sardinia | sheep | Bonelli et al. 2020 |
| MK780848 | Sardinia | sheep | Bonelli et al. 2020 |
| MK780849 | Sardinia | sheep | Bonelli et al. 2020 |
| MK780850 | Sardinia | sheep | Bonelli et al. 2020 |
| MK780851 | Sardinia | sheep | Bonelli et al. 2020 |
| MK780852 | Sardinia | cattle | Bonelli et al. 2020 |
| MK780853 | Sardinia | sheep, cattle | Bonelli et al. 2020 |
| MK780854 | Sardinia | cattle | Bonelli et al. 2020 |
| MK780855 | Sardinia | sheep | Bonelli et al. 2020 |
| MG672280 | Italy | sheep | Kinkar et al. 2018c |
| MG672281 | Italy | sheep | Kinkar et al. 2018c |
| MG672278 | Italy | sheep | Kinkar et al. 2018c |
| MG672279 | Italy | sheep | Kinkar et al. 2018c |
| MG672277 | Italy | sheep | Kinkar et al. 2018c |
| MG672136 | Italy | sheep | Kinkar et al. 2018c |
| MG672135 | Italy | human | Kinkar et al. 2018c |
| MG672134 | Italy | cattle | Kinkar et al. 2018c |
| MG672133 | Italy | cattle | Kinkar et al. 2018c |
| MG682521 | Italy | sheep | Kinkar et al. 2018a |
| MG682518 | Italy | sheep | Kinkar et al. 2018a |
| MG682517 | Italy | sheep | Kinkar et al. 2018a |
| KU925428 | Italy | cattle | Kinkar et al. 2016 |
| KU925427 | Italy | cattle | Kinkar et al. 2016 |
| KU925426 | Italy | sheep | Kinkar et al. 2016 |
| KU925425 | Italy | sheep | Kinkar et al. 2016 |
| KU925424 | Italy | sheep | Kinkar et al. 2016 |
| KU925423 | Italy | cattle | Kinkar et al. 2016 |
| MG682521 | Italy | sheep | Kinkar et al. 2018a |
| MG682522 | Italy | sheep | Kinkar et al. 2018a |
| MK806393 | Italy | wild boar | Sgroi et al. 2019 |
| KY766900 | Spain | sheep | Kinkar et al. 2017 |
| KY766896 | Spain | sheep | Kinkar et al. 2017 |
| KY766903 | Spain | sheep | Kinkar et al. 2017 |
| KY766897 | Spain | sheep | Kinkar et al. 2017 |
| KY766886 | Spain | sheep | Kinkar et al. 2017 |
| KU925413 | Spain | human | Kinkar et al. 2016 |
| KU925414 | Spain | human | Kinkar et al. 2016 |
| KU925415 | Spain | wild boar | Kinkar et al. 2016 |
| KU925416 | Spain | sheep | Kinkar et al. 2016 |
| KU925417 | Spain | sheep | Kinkar et al. 2016 |
| KU925418 | Spain | sheep | Kinkar et al. 2016 |
| KU925419 | Spain | sheep | Kinkar et al. 2016 |
| KU925420 | Spain | sheep | Kinkar et al. 2016 |
| KU925421 | Spain | pig | Kinkar et al. 2016 |
| KU925422 | Spain | goat | Kinkar et al. 2016 |
| MG672137 | Spain | human | Kinkar et al. 2018c |
| MG672139 | Spain | wild boar | Kinkar et al. 2018c |
| MG672147 | Spain | sheep | Kinkar et al. 2018c |
| MG672148 | Spain | sheep | Kinkar et al. 2018c |
| MG672149 | Spain | sheep | Kinkar et al. 2018c |
| MG672150 | Spain | sheep | Kinkar et al. 2018c |
| MG672151 | Spain | sheep | Kinkar et al. 2018c |
| MG672152 | Spain | domestic pig | Kinkar et al. 2018c |
| MG672153 | Spain | goat | Kinkar et al. 2018c |
| MG672154 | Spain | goat | Kinkar et al. 2018c |
| MG672129 | Spain | human | Kinkar et al. 2018c |
| MG682525 | Spain | sheep | Kinkar et al. 2018a |
| MG682526 | Spain | sheep | Kinkar et al. 2018a |
| MG682527 | Spain | sheep | Kinkar et al. 2018a |
| MG682528 | Spain | sheep | Kinkar et al. 2018a |
| MG682529 | Spain | human | Kinkar et al. 2018a |
| KU925351 | Turkey | sheep | Kinkar et al. 2016 |
| KU925352 | Turkey | sheep | Kinkar et al. 2016 |
| KU925353 | Turkey | sheep | Kinkar et al. 2016 |
| KU925354 | Turkey | sheep | Kinkar et al. 2016 |
| KU925355 | Turkey | cattle | Kinkar et al. 2016 |
| KU925356 | Turkey | cattle | Kinkar et al. 2016 |
| KU925357 | Turkey | cattle | Kinkar et al. 2016 |
| KU925358 | Turkey | cattle | Kinkar et al. 2016 |
| KU925359 | Turkey | cattle | Kinkar et al. 2016 |
| KU925360 | Turkey | cattle | Kinkar et al. 2016 |
| KU925361 | Turkey | cattle | Kinkar et al. 2016 |
| KU925362 | Turkey | cattle | Kinkar et al. 2016 |
| KU925363 | Turkey | cattle | Kinkar et al. 2016 |
| KU925364 | Turkey | cattle | Kinkar et al. 2016 |
| KU925365 | Turkey | cattle | Kinkar et al. 2016 |
| KU925366 | Turkey | cattle | Kinkar et al. 2016 |
| KU925367 | Turkey | cattle | Kinkar et al. 2016 |
| KU925368 | Turkey | cattle | Kinkar et al. 2016 |
| KU925369 | Turkey | cattle | Kinkar et al. 2016 |
| KU925370 | Turkey | cattle | Kinkar et al. 2016 |
| KU925371 | Turkey | cattle | Kinkar et al. 2016 |
| KU925372 | Turkey | cattle | Kinkar et al. 2016 |
| KU925373 | Turkey | cattle | Kinkar et al. 2016 |
| KU925374 | Turkey | cattle | Kinkar et al. 2016 |
| KU925375 | Turkey | cattle | Kinkar et al. 2016 |
| KU925376 | Turkey | cattle | Kinkar et al. 2016 |
| KU925377 | Turkey | cattle | Kinkar et al. 2016 |
| KU928378 | Turkey | cattle | Kinkar et al. 2016 |
| KU925379 | Turkey | cattle | Kinkar et al. 2016 |
| KU925380 | Turkey | cattle | Kinkar et al. 2016 |
| KU925381 | Turkey | sheep | Kinkar et al. 2016 |
| KU925382 | Turkey | cattle | Kinkar et al. 2016 |
| KU925383 | Turkey | cattle | Kinkar et al. 2016 |
| KU925384 | Turkey | cattle | Kinkar et al. 2016 |
| KU925385 | Turkey | sheep, cattle | Kinkar et al. 2016 |
| KU925386 | Turkey | cattle | Kinkar et al. 2016 |
| KU925387 | Turkey | cattle | Kinkar et al. 2016 |
| KU925388 | Turkey | sheep | Kinkar et al. 2016 |
| KU925389 | Turkey | sheep | Kinkar et al. 2016 |
| KU925390 | Turkey | sheep | Kinkar et al. 2016 |
| KU925391 | Turkey | sheep | Kinkar et al. 2016 |
| KU925392 | Turkey | sheep | Kinkar et al. 2016 |
| KU925393 | Turkey | sheep | Kinkar et al. 2016 |
| KU925394 | Turkey | sheep | Kinkar et al. 2016 |
| KU925395 | Turkey | sheep, cattle | Kinkar et al. 2016 |
| KU925396 | Turkey | sheep | Kinkar et al. 2016 |
| KU925397 | Turkey | sheep | Kinkar et al. 2016 |
| KU925398 | Turkey | sheep | Kinkar et al. 2016 |
| KU925399 | Turkey | sheep | Kinkar et al. 2016 |
| KU925400 | Turkey | sheep | Kinkar et al. 2016 |
| KU925401 | Turkey | sheep | Kinkar et al. 2016 |
| KU925402 | Turkey | sheep | Kinkar et al. 2016 |
| KU925403 | Turkey | sheep | Kinkar et al. 2016 |
| KU925404 | Turkey | sheep | Kinkar et al. 2016 |
| KU925405 | Turkey | sheep | Kinkar et al. 2016 |
| KU925406 | Turkey | sheep | Kinkar et al. 2016 |
| KU925407 | Turkey | sheep | Kinkar et al. 2016 |
| KU925408 | Turkey | cattle | Kinkar et al. 2016 |
| KU925409 | Turkey | cattle | Kinkar et al. 2016 |
| KU925410 | Turkey | cattle | Kinkar et al. 2016 |
| KU925411 | Turkey | cattle | Kinkar et al. 2016 |
| KU925412 | Turkey | sheep | Kinkar et al. 2016 |
| MG682536 | Turkey | sheep | Kinkar et al. 2018a |
| MG682535 | Turkey | sheep | Kinkar et al. 2018a |
| MG682530 | Turkey | cattle | Kinkar et al. 2018a |
| MG682531 | Turkey | cattle | Kinkar et al. 2018a |
| MG682532 | Turkey | cattle | Kinkar et al. 2018a |
| MG682533 | Turkey | cattle | Kinkar et al. 2018a |
| MG682534 | Turkey | sheep | Kinkar et al. 2018a |
| MG672205 | Turkey | sheep | Kinkar et al. 2018c |
| MG672172 | Turkey | sheep | Kinkar et al. 2018c |
| MG672173 | Turkey | sheep | Kinkar et al. 2018c |
| MG672174 | Turkey | sheep | Kinkar et al. 2018c |
| MG672175 | Turkey | sheep | Kinkar et al. 2018c |
| MG672176 | Turkey | sheep | Kinkar et al. 2018c |
| MG672177 | Turkey | cattle | Kinkar et al. 2018c |
| MG672178 | Turkey | cattle | Kinkar et al. 2018c |
| MG672179 | Turkey | cattle | Kinkar et al. 2018c |
| MG672180 | Turkey | cattle | Kinkar et al. 2018c |
| MG672181 | Turkey | cattle | Kinkar et al. 2018c |
| MG672182 | Turkey | cattle | Kinkar et al. 2018c |
| MG672183 | Turkey | cattle | Kinkar et al. 2018c |
| MG672184 | Turkey | cattle | Kinkar et al. 2018c |
| MG672185 | Turkey | cattle | Kinkar et al. 2018c |
| MG672186 | Turkey | cattle | Kinkar et al. 2018c |
| MG672187 | Turkey | cattle | Kinkar et al. 2018c |
| MG672188 | Turkey | cattle, sheep | Kinkar et al. 2018c |
| MG672189 | Turkey | cattle | Kinkar et al. 2018c |
| MG672127 | Turkey | sheep | Kinkar et al. 2018c |
| MG672190 | Turkey | sheep | Kinkar et al. 2018c |
| MG672191 | Turkey | sheep | Kinkar et al. 2018c |
| MG672192 | Turkey | sheep | Kinkar et al. 2018c |
| MG672193 | Turkey | sheep | Kinkar et al. 2018c |
| MG672194 | Turkey | sheep | Kinkar et al. 2018c |
| MG672124 | Turkey | sheep | Kinkar et al. 2018c |
| MG672195 | Turkey | sheep | Kinkar et al. 2018c |
| MG672196 | Turkey | sheep | Kinkar et al. 2018c |
| MG672197 | Turkey | sheep | Kinkar et al. 2018c |
| MG672198 | Turkey | sheep | Kinkar et al. 2018c |
| MG672199 | Turkey | sheep | Kinkar et al. 2018c |
| MG672200 | Turkey | sheep | Kinkar et al. 2018c |
| MG672201 | Turkey | sheep | Kinkar et al. 2018c |
| MG672202 | Turkey | cattle | Kinkar et al. 2018c |
| MG672203 | Turkey | sheep | Kinkar et al. 2018c |
| MG672204 | Turkey | sheep | Kinkar et al. 2018c |
| MG672205 | Turkey | sheep | Kinkar et al. 2018c |
| KT716269 | Tunisia | antelope | Boufana et al. 2015a |
| KT716270 | Tunisia | antelope | Boufana et al. 2015a |
| KT001399 | Tunisia | dog | Boufana et al. 2015b |
| KT001400 | Tunisia | dog, jackal | Boufana et al. 2015b |
| KT001401 | Tunisia | dog | Boufana et al. 2015b |
| KT001402 | Tunisia | dog | Boufana et al. 2015b |
| KT001403 | Tunisia | dog | Boufana et al. 2015b |
| KT001404 | Tunisia | dog | Boufana et al. 2015b |
| KT001406 | Tunisia | dog | Boufana et al. 2015b |
| KM014644 | Tunisia | sheep | Boufana et al. 2014 |
| KM014643 | Tunisia | human | Boufana et al. 2014 |
| KM014642 | Tunisia | cattle | Boufana et al. 2014 |
| KM014641 | Tunisia | wild boar | Boufana et al. 2014 |
| KM014640 | Tunisia | dog | Boufana et al. 2014 |
| KM014639 | Tunisia | cattle | Boufana et al. 2014 |
| KM014638 | Tunisia | dog | Boufana et al. 2014 |
| KM014637 | Tunisia | human | Boufana et al. 2014 |
| KM014636 | Tunisia | dog | Boufana et al. 2014 |
| KM014635 | Tunisia | dog | Boufana et al. 2014 |
| KM014634 | Tunisia | cattle | Boufana et al. 2014 |
| KM014633 | Tunisia | camel | Boufana et al. 2014 |
| KM014632 | Tunisia | wild boar | Boufana et al. 2014 |
| KM014631 | Tunisia | human | Boufana et al. 2014 |
| KM014630 | Tunisia | dog | Boufana et al. 2014 |
| KM014629 | Tunisia | human | Boufana et al. 2014 |
| KM014628 | Tunisia | sheep | Boufana et al. 2014 |
| KM014627 | Tunisia | human | Boufana et al. 2014 |
| KM014626 | Tunisia | donkey | Boufana et al. 2014 |
| KM014625 | Tunisia | donkey | Boufana et al. 2014 |
| KM014624 | Tunisia | goat | Boufana et al. 2014 |
| KM014623 | Tunisia | wild boar | Boufana et al. 2014 |
| KM014622 | Tunisia | wild boar | Boufana et al. 2014 |
| KM014621 | Tunisia | human | Boufana et al. 2014 |
| KM014620 | Tunisia | cattle | Boufana et al. 2014 |
| KM014619 | Tunisia | camel | Boufana et al. 2014 |
| KM014618 | Tunisia | donkey | Boufana et al. 2014 |
| KM014617 | Tunisia | cattle | Boufana et al. 2014 |
| KM014616 | Tunisia | goat | Boufana et al. 2014 |
| KM014615 | Tunisia | cattle | Boufana et al. 2014 |
| KM014614 | Tunisia | sheep | Boufana et al. 2014 |
| KM014613 | Tunisia | sheep | Boufana et al. 2014 |
| KM014612 | Tunisia | cattle | Boufana et al. 2014 |
| KM014611 | Tunisia | wild boar | Boufana et al. 2014 |
| KM014610 | Tunisia | dog | Boufana et al. 2014 |
| KM014609 | Tunisia | camel, sheep | Boufana et al. 2014 |
| KM014608 | Tunisia | sheep | Boufana et al. 2014 |
| KM014607 | Tunisia | wild boar | Boufana et al. 2014 |
| KM014606 | Tunisia | wild boar, camel, cattle, goat, jackal, dog, sheep, human | Boufana et al. 2014 |
| MG672155 | Tunisia | sheep | Kinkar et al. 2018c |
| MG672156 | Tunisia | sheep | Kinkar et al. 2018c |
| MG672157 | Tunisia | sheep | Kinkar et al. 2018c |
| MG672158 | Tunisia | sheep | Kinkar et al. 2018c |
| MG672159 | Tunisia | sheep | Kinkar et al. 2018c |
| MG672160 | Tunisia | sheep | Kinkar et al. 2018c |
| MG672161 | Tunisia | sheep | Kinkar et al. 2018c |
| MG672162 | Tunisia | sheep | Kinkar et al. 2018c |
| MG672163 | Tunisia | sheep | Kinkar et al. 2018c |
| MG672164 | Tunisia | sheep | Kinkar et al. 2018c |
| MG672165 | Tunisia | sheep | Kinkar et al. 2018c |
| MG672166 | Tunisia | sheep | Kinkar et al. 2018c |
| MG672167 | Tunisia | sheep | Kinkar et al. 2018c |
| MG672168 | Tunisia | sheep, cattle | Kinkar et al. 2018c |
| MG672169 | Tunisia | sheep, human | Kinkar et al. 2018c |
| MG672170 | Tunisia | cattle | Kinkar et al. 2018c |
| MG672171 | Tunisia | cattle | Kinkar et al. 2018c |
| MG672264 | Tunisia | human | Kinkar et al. 2018c |
| MG672265 | Tunisia | human | Kinkar et al. 2018c |
| MG672266 | Tunisia | human | Kinkar et al. 2018c |
| MG672267 | Tunisia | human | Kinkar et al. 2018c |
| MG672268 | Tunisia | human | Kinkar et al. 2018c |
| MG672269 | Tunisia | human | Kinkar et al. 2018c |
| MG672270 | Tunisia | human | Kinkar et al. 2018c |
| MG672271 | Tunisia | human | Kinkar et al. 2018c |
| MG672272 | Tunisia | human | Kinkar et al. 2018c |
| MG672273 | Tunisia | human | Kinkar et al. 2018c |
| MG672274 | Tunisia | human | Kinkar et al. 2018c |
| MG672275 | Tunisia | human | Kinkar et al. 2018c |
| MG672276 | Tunisia | human | Kinkar et al. 2018c |
